# Purpura Fulminans in a Patient With Cirrhosis and Methicillin-Sensitive Staphylococcus aureus (MSSA) Bacteremia: Diagnostic Challenges in Coagulopathy

**DOI:** 10.7759/cureus.106365

**Published:** 2026-04-03

**Authors:** Donnell D White, Katherine Blaise, Taylor L Dartez, Andrew Carbajal, Melanie Bienvenue, Christopher Wexler, Christopher Chedid

**Affiliations:** 1 School of Medicine, Louisiana State University Health Sciences Center - New Orleans, New Orleans, USA; 2 Department of Internal Medicine, Louisiana State University Health Sciences Center - Lafayette, Lafayette, USA

**Keywords:** acute infectious purpura fulminans, alcoholic cirrhosis, alcoholic liver cirrhosis, sepsis-associated disseminated intravascular coagulation (dic), staph aureus bacteremia

## Abstract

Disseminated intravascular coagulation (DIC) is a critical complication of systemic illness, and its diagnosis can be especially challenging in patients with cirrhosis, where baseline liver-related coagulopathy may mask the development of superimposed consumptive coagulopathy. In the setting of sepsis and liver dysfunction, DIC may progress to purpura fulminans (PF), a rare, life-threatening condition characterized by dermal necrosis and microvascular thrombosis. We present the case of a 54-year-old man with alcoholic cirrhosis and untreated hepatitis C who developed DIC and PF following methicillin-sensitive *Staphylococcus aureus* (MSSA) bacteremia and toxic shock syndrome (TSS). Despite treatment with antibiotics, transfusions, intravenous immunoglobulin (IVIG), and hemodialysis, he developed multiorgan failure. This case highlights that in patients with sepsis and cirrhosis, acutely worsening coagulation parameters, falling fibrinogen, and evolving purpuric or necrotic skin lesions should not be attributed to liver disease alone but should instead prompt urgent evaluation for DIC and PF. The early recognition of this distinction is essential to guide timely multidisciplinary intervention in this rare but devastating complication.

## Introduction

Disseminated intravascular coagulation (DIC) is a complex and acquired syndrome characterized by the widespread intravascular activation of the coagulation cascade, leading to widespread clot formation and the subsequent consumption of clotting factors [1]. Tissue factors enter circulation due to trauma-related endothelial damage, certain cancer treatments, bacterial endotoxins, or cytokine exposure, activating coagulation factors and the formation of thrombin and fibrin, resulting in circulating clots. Coagulation inhibitors are subsequently consumed, causing excessive bleeding [2]. Around 30%-50% of patients with sepsis develop DIC, whereas the incidence of DIC is around 10% in patients with solid tumors, trauma, or obstetric complications [3].

Although DIC is well recognized in patients with sepsis, its diagnosis is substantially more difficult in those with cirrhosis, where baseline liver-related coagulopathy may mask evolving consumptive coagulopathy. In advanced cases, DIC can progress to purpura fulminans (PF), a rare and life-threatening condition characterized by dermal necrosis and microvascular thrombosis [4]. The most common subtype is acute infectious PF caused by an acquired protein C deficiency, as coagulation factors are consumed in consumptive coagulopathy associated with severe infections. *Neisseria meningitidis* is the most common culprit due to both its endotoxin production and ability to alter endothelial interaction with protein C. Reports linked to methicillin-sensitive *Staphylococcus aureus* (MSSA) toxic shock syndrome (TSS) are uncommon.

We describe a patient with alcoholic cirrhosis, untreated hepatitis C, MSSA bacteremia, and toxic shock syndrome who developed DIC and PF, illustrating both the diagnostic difficulty of distinguishing DIC from decompensated liver disease and the importance of recognizing progressive purpuric skin lesions and falling fibrinogen as warning signs of superimposed consumptive coagulopathy.

## Case presentation

A 54-year-old man with a past medical history significant for alcoholic cirrhosis, untreated hepatitis C, and chronic tobacco use presented with complaints of progressive weakness, epistaxis, acute kidney injury (AKI), and the worsening of his model for end-stage liver disease-sodium (MELD-Na) score from 21 to 40. On presentation, heart rate, blood pressure, and temperature were normal, but his respiratory rate was elevated, and studies demonstrated leukocytosis (Table 1). Given his underlying cirrhosis, the initial differential diagnosis included decompensated liver disease with liver-related coagulopathy, hepatorenal syndrome, and superimposed infection. Blood cultures revealed methicillin-sensitive *Staphylococcus aureus* (MSSA), which further compounded his deteriorating condition, and he was started on oxacillin. His urinalysis showed white blood cells, red blood cells, and protein, so a diagnosis other than hepatorenal syndrome had to be explored. His kidney injury was initially suspected to be due to acute interstitial nephritis (AIN) caused by nonsteroidal anti-inflammatory drug (NSAID) use, but it progressively worsened despite the discontinuation of the medication, eventually necessitating hemodialysis (Table 1).

**Table 1:** Serial laboratory values during hospitalization, highlighting disseminated intravascular coagulation, cytopenias, hemolysis, and organ dysfunction in this case of purpura fulminans. BUN, blood urea nitrogen; LDH, lactate dehydrogenase; ALP, alkaline phosphatase; ALT, alanine transaminase; AST, aspartate transaminase; CK, creatine kinase; WBC, white blood cell; Hgb, hemoglobin; HCT, hematocrit; PT, prothrombin time; INR, international normalized ratio; APTT, activated partial thromboplastin time; FDP, fibrin degradation product

Variable	Presentation	Day 2	Day 3	Day 4	Day 5	Day 6	Day 7
BUN (8.4-25.7 mg/dL)	57.1	65.2	83.9	89	73	18	11
Creatinine (0.73-1.18 mg/dL)	3.99	4.16	4.94	5.62	4.5	1.9	1.3
Total bilirubin (*≤*1.5 mg/dL)	8.2	9.3	7.6	6.9	6.6	9	9.7
Direct bilirubin (0.0 to <0.5 mg/dL)	-	5.3	4.7	4.4	-	-	-
LDH (125-220 U/L)	-	177	-	-	336	-	-
Haptoglobin (30-250 mg/dL)	-	82	-	-	43	-	-
Lactate (0.5-2.2 mmol/L)	-	-	-	6.9	4.5	2.8	4.1
ALP (40-150 units/L)	84	74	91	67	50	65	99
ALT (0-55 units/L)	20	20	24	28	28	37	48
AST (5-34 units/L)	43	50	68	81	76	88	113
CK (30-200 U/L)	81	-	-	-	954	-	-
WBC (4.50-11.50*×*10^3^/mcL)	13.54	14.6	13.3	13.47	15.06	18.51	20.67
Hgb (14.0-18.0 g/dL)	10.5	10.1	8.8	5.9	7	7.6	6.5
HCT (42.0%-52.0%)	30.6	28.7	26.2	17.2	22	23.1	22
Platelets (130-400*×*10^3^/mcL)	44	39	53	33	48	53	50
D-dimer (0-0.5 μg/mL)	-	19.77	-	-	-	-	-
PT (9.0-12.5 seconds)	30.4	32.9	33	50.8	18.3	19.9	25.4
INR (0.8-1.2)	2.8	3.1	3.1	5.4	1.7	1.9	2.5
APTT (21.0-32.0 seconds)	55.7	60.9	53.8	64.1	38.9	43.9	55.3
Fibrinogen (182-400 mg/dL)	-	92	78	<60	146	96	89
Protein C activity (72%-160%)	-	-	-	-	-	-	14
Protein S activity (65%-150%)	-	-	-	-	-	-	30
FDP (<5 μg/mL)	-	>20	-	-	-	-	-
Factor V Leiden	-	-	-	-	-	-	Negative
Factor VIII (59%-19%)	-	110	128	-	-	-	-
Ammonia (18-72.0 μmol/L)	31	-	-	-	-	-	-

**Figure 1:**
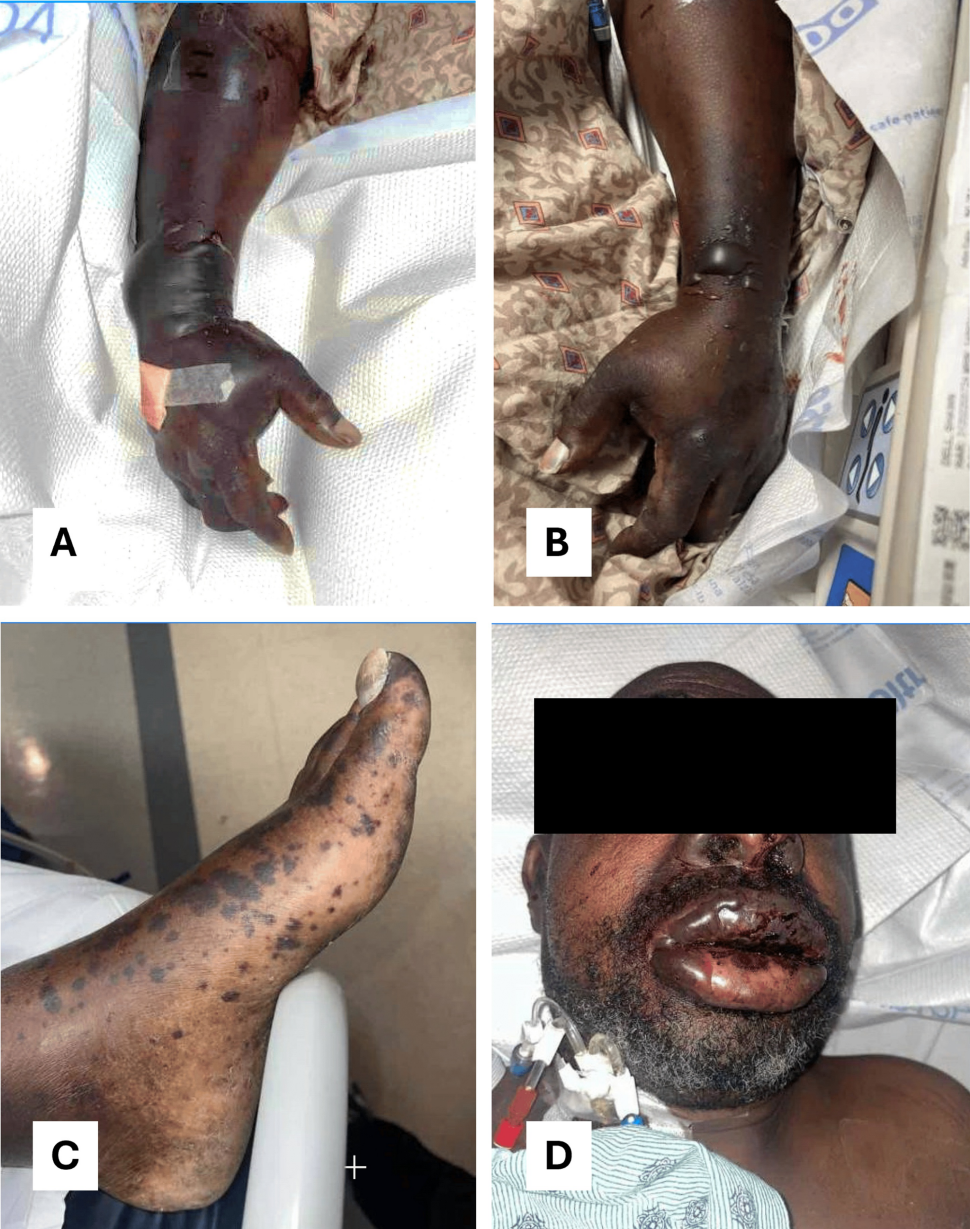
Clinical photographs demonstrating cutaneous and mucosal manifestations of purpura fulminans. Hemorrhagic bullae involving the upper extremities and feet in the setting of purpura fulminans development and progression (A-C). Mucosal swelling with associated bullae formation (D).

As his condition acutely worsened, the patient became less responsive and hypothermic, and he developed skin sloughing with purpuric blistering lesions on his extremities and trunk (Figure 1). Stevens-Johnson syndrome was considered, given the skin sloughing, but the presence of thrombotic vasculopathy on biopsy, progressive consumptive coagulopathy, and purpuric necrotic lesions favored purpura fulminans, while the concurrent MSSA bacteremia and clinical deterioration supported staphylococcal toxic shock syndrome as the precipitating infectious trigger. Clindamycin and intravenous immunoglobulin (IVIG) were administered to treat TSS. A skin biopsy was obtained and revealed thrombotic vasculopathy per pathology, thus confirming the diagnosis of purpura fulminans (PF). Also, at this point, his creatinine was significantly elevated, and he continued to require hemodialysis (Table 1). The differential for his AKI was expanded to include acute tubular necrosis (ATN) due to thrombotic microangiopathy and sepsis. Although acute interstitial nephritis was initially considered because of prior NSAID exposure, the progressive course despite discontinuation, in conjunction with sepsis, shock physiology, severe coagulopathy, and only rare schistocytes on peripheral smear, made sepsis-associated ATN with possible thrombotic microangiopathic contribution more likely than isolated drug-induced nephritis or a primary thrombotic microangiopathy.

Throughout the hospitalization, his clinical course and coagulation studies became increasingly consistent with progressive disseminated intravascular coagulation (DIC), and his International Society on Thrombosis and Haemostasis (ISTH) score remained above 5 throughout hospitalization. His international normalized ratio (INR) rose significantly, reflective of both worsening liver dysfunction and consumption coagulopathy. Interestingly, his fibrinogen levels declined to critically low levels, consistent with a consumptive coagulopathy pattern seen in DIC, which can be distinguished from normal or near-normal fibrinogen seen in stable cirrhosis (Table 1). Factor VIII (FVIII) level was elevated, which would usually suggest coagulopathy due to liver disease rather than DIC; however, this was difficult to interpret in the setting of sepsis, as factor VIII is also an acute-phase reactant. Hemoglobin levels dropped below the threshold requiring transfusion, and he had continued epistaxis and subcutaneous ecchymosis around his nares, midface, and tongue, suggestive of ongoing bleeding. Platelets remained critically low, and the peripheral smear showed rare schistocytes, suggesting a minimal to minor component of microangiopathic hemolytic anemia, which was consistent with DIC (Table 1). During his hospital course, he continued oxacillin and clindamycin for MSSA and TSS. He was switched to Zosyn due to his rapid development of skin necrosis and wet gangrene. Finally, he was transitioned to cefazolin and ertapenem for salvage therapy. No source of the persistent bacteremia was found in echocardiography and advanced imaging.

The patient’s severe DIC and PF were likely multifactorial, as the patient had cirrhosis, sepsis due to MSSA bacteremia, and suspected toxic shock syndrome. Despite aggressive management with transfusions of blood products, including platelets, fresh frozen plasma, and cryoprecipitate, along with broad-spectrum antibiotics and intravenous fluid (IVF) targeting sepsis, the patient’s condition continued to decline. The coagulopathy led to further complications, including multiorgan failure and, ultimately, cardiac arrest.

## Discussion

**Figure 2:**
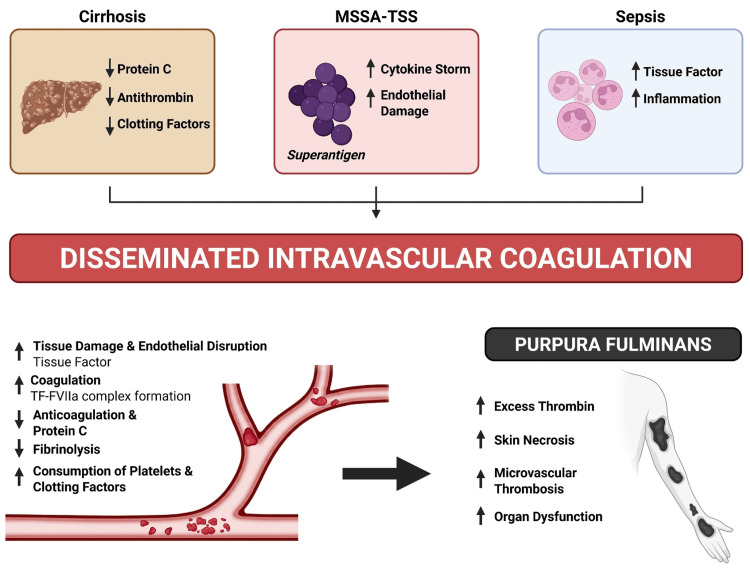
Proposed mechanism of purpura fulminans in this case. Cirrhosis, MSSA toxic shock syndrome (TSS), and sepsis likely worked in sync to produce a profoundly prothrombotic and dysregulated hemostatic state. Reduced hepatic synthesis of key anticoagulant proteins, together with superantigen-driven cytokine release, endothelial injury, and tissue factor upregulation, likely promoted disseminated intravascular coagulation with excess thrombin generation, consumptive coagulopathy, impaired fibrinolysis, and widespread microvascular thrombosis. These intersecting processes ultimately resulted in skin necrosis, organ dysfunction, and the clinical syndrome of purpura fulminans. Image created with BioRender. MSSA, methicillin-sensitive *Staphylococcus aureus*; TF-FVIIa, tissue factor-factor VIIa

Early identification and intervention in DIC are crucial, particularly in patients with liver dysfunction and sepsis, both of which independently increase the risk of coagulopathy. Initial signs of DIC often include petechiae, bleeding from catheter sites, and abnormal coagulation markers such as elevated D-dimer, prolonged prothrombin time (PT)/INR, and low fibrinogen (Figure 2). DIC may also be associated with microangiopathic hemolytic anemia, although this is often less pronounced than in primary thrombotic microangiopathies such as thrombotic thrombocytopenic purpura. Screening with the International Society on Thrombosis and Haemostasis (ISTH) scoring system is appropriate when clinical suspicion for DIC is high, using platelet count, D-dimer, PT, and fibrinogen level [5]. In septic shock, both JAAM-DIC and ISTH overt-DIC scores have shown utility for diagnosing DIC, whereas the sepsis-induced coagulopathy score appears less specific [6,7].

In patients with liver dysfunction, distinguishing DIC from liver-related coagulopathy is particularly challenging because both conditions may present with elevated INR, thrombocytopenia, and bleeding. In this case, diagnostic uncertainty was significant, as advanced cirrhosis could explain several baseline hemostatic abnormalities. However, the rapid clinical deterioration, persistent MSSA sepsis, critically low fibrinogen, progressive purpuric skin necrosis, and biopsy-demonstrated thrombotic vasculopathy supported superimposed DIC with purpura fulminans rather than liver disease alone. No single laboratory test definitively established this distinction. Although factor VIII has historically been proposed as a discriminator, its interpretation here was limited by its role as an acute-phase reactant in sepsis. Thus, the diagnosis depended on the overall clinical context, dynamic laboratory trends, and histopathologic confirmation rather than any isolated marker (Figure 2).

Factor VIII (FVIII) has historically been proposed as a tool to help distinguish DIC from liver-related coagulopathy, as FVIII is often reduced in DIC and normal or elevated in liver disease [8]. However, more recent data suggest that FVIII does not reliably differentiate these conditions, particularly in patients with liver disease, and its interpretation is further limited in sepsis because it behaves as an acute-phase reactant [7,9]. Hepatic dysfunction may also worsen DIC by reducing the production of endogenous anticoagulants such as protein C and antithrombin while increasing prothrombotic factors, including von Willebrand factor and FVIII [10]. Together, these overlapping abnormalities highlight the need for close clinical monitoring and the careful interpretation of evolving laboratory trends in patients with decompensated liver disease.

Purpura fulminans is a rare but often fatal manifestation of DIC caused by the uncontrolled activation of coagulation in the microvasculature, resulting in dermal necrosis and hemorrhagic skin lesions. These lesions are often painful and rapidly progressive and should prompt urgent intervention. Infection-related PF caused by *Staphylococcus aureus* (*S. aureus*) is rare, although a limited number of cases have been reported, particularly in the setting of toxic shock syndrome [11]. Management centers on aggressive supportive care and the prompt treatment of the underlying trigger. Despite broad-spectrum antibiotics and intensive supportive measures, sepsis-associated PF carries a mortality rate of approximately 43% [12]. Protein C concentrate has shown potential benefit in infection-related PF [11], while corticosteroids may have a role in select cases, and the early surgical debridement of necrotic tissue has been associated with reduced mortality [13,14].

Both MSSA toxic shock syndrome and meningococcal sepsis share a pathophysiological basis in toxin-mediated massive inflammatory responses: superantigens in *S. aureus* trigger widespread T-cell activation and cytokine release, while meningococcal endotoxin (lipopolysaccharide) activates the innate immune system and coagulation cascades [15-17]. However, these mechanisms diverge in their effects on the coagulation system. Meningococcal endotoxin has a direct and profound effect on the coagulation cascade, leading to disseminated intravascular coagulation and severe acquired protein C deficiency, which is central to the pathogenesis of purpura fulminans [16]. Meningococcus also uniquely disrupts endothelial protein C receptor (EPCR) function via ADAM10-mediated shedding, further impairing protein C activation and promoting microvascular thrombosis [16,17].

## Conclusions

This case highlights the diagnostic and management challenges of distinguishing DIC from cirrhosis-associated coagulopathy in a patient with advanced liver disease and severe sepsis. It underscores the importance of integrating the full clinical picture, including dynamic laboratory trends, structured diagnostic scoring, and evolving cutaneous findings, rather than relying on any single marker alone. In addition, this case draws attention to purpura fulminans as a rare but devastating manifestation associated with MSSA toxic shock syndrome, emphasizing the need for early recognition, the prompt treatment of the infectious trigger, and aggressive multidisciplinary supportive care.
